# Gene Transcription Changes in Asthmatic Chronic Rhinosinusitis with Nasal Polyps and Comparison to Those in Atopic Dermatitis

**DOI:** 10.1371/journal.pone.0011450

**Published:** 2010-07-06

**Authors:** Douglas A. Plager, Jane C. Kahl, Yan W. Asmann, Allan E. Nilson, John F. Pallanch, Oren Friedman, Hirohito Kita

**Affiliations:** 1 Allergic Diseases Research Laboratory, Mayo Clinic, Rochester, Minnesota, United States of America; 2 Division of Biomedical Statistics and Informatics, Mayo Clinic, Rochester, Minnesota, United States of America; 3 Department of Otorhinolaryngology, Mayo Clinic, Rochester, Minnesota, United States of America; Massachusetts General Hospital/Harvard University, United States of America

## Abstract

**Background:**

Asthmatic chronic rhinosinusitis with nasal polyps (aCRSwNP) is a common disruptive eosinophilic disease without effective medical treatment. Therefore, we sought to identify gene expression changes, particularly those occurring early, in aCRSwNP. To highlight expression changes associated with eosinophilic epithelial inflammation, we further compared the changes in aCRSwNP with those in a second eosinophilic epithelial disease, atopic dermatitis (AD), which is also closely related to asthma.

**Methods/Principal Findings:**

Genome-wide mRNA levels measured by exon array in both nasosinus inflamed mucosa and adjacent polyp from 11 aCRSwNP patients were compared to those in nasosinus tissue from 17 normal or rhinitis subjects without polyps. Differential expression of selected genes was confirmed by qRT-PCR or immunoassay, and transcription changes common to AD were identified. Comparison of aCRSwNP inflamed mucosa and polyp to normal/rhinitis tissue identified 447 differentially transcribed genes at ≥2 fold-change and adjusted p-value<0.05. These included increased transcription of chemokines localized to chromosome 17q11.2 (CCL13, CCL2, CCL8, and CCL11) that favor eosinophil and monocyte chemotaxis and chemokines (CCL18, CCL22, and CXCL13) that alternatively-activated monocyte-derived cells have been shown to produce. Additional transcription changes likely associated with Th2-like eosinophilic inflammation were prominent and included increased IL1RL1 (IL33 receptor) and EMR1&3 and decreased CRISP2&3. A down-regulated PDGFB-centric network involving several smooth muscle-associated genes was also implicated. Genes at 17q11.2, genes associated with alternative activation or smooth muscle, and the IL1RL1 gene were also differentially transcribed in AD.

**Conclusions/Significance:**

Our data implicate several genes or gene sets in aCRSwNP and eosinophilic epithelial inflammation, some that likely act in the earlier stages of inflammation. The identified gene expression changes provide additional diagnostic and therapeutic targets for aCRSwNP and other eosinophilic epithelial diseases.

## Introduction

Chronic rhinosinusitis (CRS) is one of the most prevalent chronic diseases in the United States, afflicting about 10% of the population [1], [2]. Coexisting pathologies often include asthma and nasal polyps. Because there are not FDA-approved pharmaceutical interventions to treat CRS, nasal polyposis frequently leads to surgical intervention to clear the nasosinus passages, and repeat endoscopic sinus surgery is often required. Asthma is equally prominent and socioeconomically detrimental.

Asthmatic CRS (aCRS), and other allergic diseases such as atopic dermatitis (AD), which has an asthma comorbidity of about 30%, are generally associated with Th2-like eosinophilic inflammation [3], [4], [5]. As end-stage effector cells, eosinophils contribute to epithelial damage [6]; although, recent studies suggest a role for eosinophils in earlier stages of allergic disease [7], [8], [9]. Resident epithelial and mast cells and other tissue-infiltrating cell types, particularly dendritic and T helper cells, also contribute to eosinophilic inflammation. Gene expression changes preceding chronic eosinophilic inflammation are not fully understood, but intervening early in an eosinophilic inflammatory response to minimize downstream inflammation is appealing.

High-density oligonucleotide arrays, often referred to as microarrays, are a popular screening method for gene transcription changes associated with disease pathology. A literature search for articles involving microarray analysis of sinusitis or nasal polyps identified 13 relevant articles; three included large-scale gene transcription analysis of tissue from patients with CRS, asthma, and nasal polyps [10], [11], [12]. However, several discordant gene transcription changes were reported by Liu et al. [10] compared to Stankovic et al. [11]. Also, none of these studies sought to analyze gene transcription changes in adjacent inflamed nasosinus mucosa, which may help identify gene transcription changes preceding more pronounced inflammation. Furthermore, accessibility to well-preserved human asthmatic lung tissue for gene expression analyses is limited, with only one published genome-wide transcription study identified [13]. Interestingly, a recent report indicated that nasal tissue could be a reasonable surrogate for lung tissue [14]. Therefore, we studied both nasosinus polyps and adjacent inflamed mucosa from young aCRSwNP subjects to identify gene expression changes, particularly early changes, in the inflammatory process of this and other eosinophilic conditions.

## Materials and Methods

### Research Subjects and Tissue Collection

Ethics Statement: All specimens were collected at the Mayo Clinic - Rochester and after obtaining informed written consent under a Mayo Clinic - Rochester Institutional Review Board-approved protocol. Twelve aCRS subjects (11 with nasal polyps) provided inflamed nasosinus mucosa (11 samples) and adjacent polyp (10 samples) or a small mixed mucosa and polyp specimen (1 sample). Inflamed mucosa or polyp was from the middle meatus or anterior ethmoid cavity. Inclusion criteria for aCRS subjects included two or more of the following symptoms for >12 weeks: nasal obstruction, nasal discharge, hyposmia, and nasal cavity purulence. Computed tomography (CT) confirmation of basement membrane thickening, endoscopic assessment of nasosinus passages, and concurrent physician-diagnosed asthma together with objective evidence for airway hyperreactivity or airway reversibility were also required. Among the normal or rhinitis subjects without nasal polyps (n = 17), 4 had physician-diagnosed allergic rhinitis (AR), 2 had suspected AR, 1 had suspected vasomotor rhinitis, and 10 had no signs of nasosinus disease, allergy, or atopy. Normal and rhinitis tissue was collected from the inferior turbinate or uncinate process. All 4 AR subjects chose to donate tissue outside of their known allergy season(s). General exclusion criteria included smoking, history of immunodeficiency or cystic fibrosis, pregnancy or lactation, overt bacterial or viral infection, and use of antibiotics, systemic glucocorticoids, or allergy immunotherapy 7 days, 3 months, or 1 year, respectively, before tissue donation. Rhinitis or aCRS subjects also stopped intranasal and oral medications ≥4 days and inhaled medications ≥1 day before tissue donation. All subjects refrained from using non-prescribed medications ≥4 days before tissue donation. Pre-operative intranasal preparation included topical 4% cocaine and injected 1% lidocaine with 1/100,000 epinephrine. Tissue was immediately placed in 4 mL of room temperature RNAlater for 2 minutes with mixing, transported on ice, held overnight at 4°C, and placed at −20°C until total RNA isolation.

### Total RNA Isolation

After removal from RNAlater, each specimen was frozen in liquid nitrogen, placed in ice-cold TRIzol (Invitrogen, Carlsbad, CA), and immediately shattered and homogenized with a Polytron homogenizer (Brinkmann Instruments, Westbury, NY). Total RNA was isolated according to the manufacturer's protocol for TRIzol and further purified using the RNeasy Mini kit (Qiagen, Valencia, CA). Purified total RNA was supplied to the Mayo Microarray Core facility, assessed for integrity using the Agilent Bioanalyzer 2100 (Agilent Technologies, Palo Alto, CA), and used for exon array analyses. To compensate for potential variability among exon array analyses, total RNA was isolated from and analyzed in batches comprised of different specimen types. Excess total RNA was stored at −80°C.

### Exon Array Hybridization

Following the Affymetrix GeneChip® Whole Transcript Sense Target Labeling Assay (Affymetrix, Santa Clara, CA), total RNA was depleted of ribosomal RNA and converted into double-stranded cDNA using random hexamers tagged with a T7 promoter sequence. This cDNA was used as template for T7 RNA Polymerase amplification into antisense cRNA. The cRNA was converted into single-stranded sense DNA with incorportated dUTP, fragmented at the dUTP sites, and end-labeled with biotin. Hybridizations to Affymetrix GeneChip® Human Exon 1.0 ST Arrays were performed overnight at 45°C. Arrays were washed, stained with phycoerythrin-labeled streptavidin, and scanned in the Affymetrix GeneChip® Scanner. The resulting image files were analyzed using Affymetrix Microarray Suite 5.1 to generate .CEL files containing the probe level intensities and the x, y coordinates of each probe on the array.

### Exon Array Data Analysis

The 39 exon array .CEL files (deposited at NCBI Gene Expression Omnibus (GEO)) were imported using Partek® Genomics Suite™ software (Partek Incorporated, St. Louis, MO) and normalized using default RMA settings. Transcript expression levels were calculated using the well-established “core” set of exons for 21,980 distinct genes. Comparisons between sample groups were performed using GeneSpring GX 9.0 software (Silicon Genetics, Redwood City, CA). Molecular network analyses of the identified differentially transcribed genes used Ingenuity Pathways Analysis software (Ingenuity Systems, Mountain View, CA).

### Quantitative RT-PCR

Excess total RNAs stored at −80°C and from the same preparations used to generate exon array data were used for qRT-PCR. Reverse transcription used 2 µg of total RNA and the SuperScript VILO cDNA synthesis kit (Invitrogen). Resulting cDNA samples were used as PCR template in conjunction with SYBR GreenER qPCR Supermix (Invitrogen). All PCR primer pairs were from SABiosciences (Frederick, MD). Standard curves for housekeeping genes (B2M, ACTB, and GAPDH) and target genes (CCL13, CCL18, CRISP3, EMR3, and IL1RL1) were generated using eight two-fold dilutions of cDNA produced from a pooled sample of total RNA from two normal and one aCRSwNP polyp specimens. Individual cDNA samples were used in triplicate PCRs over a 10-fold dilution range and these were performed twice. Threshold cycle numbers for the various PCRs were compared to the appropriate standard curve to calculate a relative quantity of starting cDNA for each housekeeping gene and each target gene within each individual sample tested. Ratios of target gene quantity to each of the three housekeeping gene quantities for each sample were subsequently calculated. Because using data for each of the individual housekeeping genes provided proportionally similar ratio results, an average target gene to housekeeping genes ratio was calculated for each sample tested.

### Charcot-Leyden Crystal (CLC) protein Competitive Radioimmunoprecipitation Assay

Nasal lavage fluid or secretions were collected under Mayo IRB-approved protocols from normal, AR, and CRS subjects distinct from those donating nasosinus tissue for exon array analyses. Recombinant human CLC protein (kindly provided by SJ Ackerman, The University of Illinois at Chicago) labeled with ^125^I was mixed with 2-fold dilutions of unlabeled, purified recombinant human CLC protein, for standard curve generation, or with test sample and with anti-human CLC rabbit polyclonal antibody (generated in the Mayo Allergic Diseases Research Laboratory) overnight at 4°C. Burro anti-rabbit IgG antibody was added and the mixture was left at room temperature for 3 hr and then centrifuged at 1000×g for 15 min. Supernatant was carefully drained away for 1 min and the immunoprecipitated pellet was counted for radioactivity.

### Comparison of Atopic Dermatitis (AD) and aCRSwNP Array Data

AD array data sets from Olsson et al. (GSE6012 NCBI Gene Expression Omnibus (GEO); lesional AD skin, n = 10, and normal skin, n = 10) [15] and from our group (GSE5667 NCBI GEO; minimally lesional AD skin, n = 6, and nearby visibly nonlesional AD skin, n = 6, and normal skin, n = 5) [16] were imported separately into GeneSpring GX 9.0. After an initial low stringency comparison between AD and normal samples within each data set (fold change (Fc)≥1.4 and unpaired nonparametric Mann-Whitney U test, p≤0.22), subsequent analyses for differentially transcribed genes between AD and normal samples at Fc≥2.0 and adjusted p-value≤0.1 (unpaired nonparametric test with Benjamini-Hochberg False Discovery Rate adjustment) were performed. A union set (i.e. a combined list) from the two AD to normal skin comparisons was generated, and the intersection of this union set with the list of differentially transcribed genes from the comparison of aCRSwNP to normal/rhinitis nasosinus tissue (Fc≥2.0 and adjusted p-value≤0.05) was generated based on identical Gene Symbols or RefSeq Transcript IDs using Microsoft Office Access 2003 software to identify the differentially transcribed genes common to AD and aCRSwNP.

### Immunohistochemical Staining for CD11c

CD11c staining was performed using mouse anti-human CD11c (BD Pharmingen, San Diego, CA) and the UltraVision LP Detection kit (Lab Vision Corp., Fremont, CA) according to the manufacturer's instructions on frozen nasosinus tissue sections.

## Results

**Figure 1:**
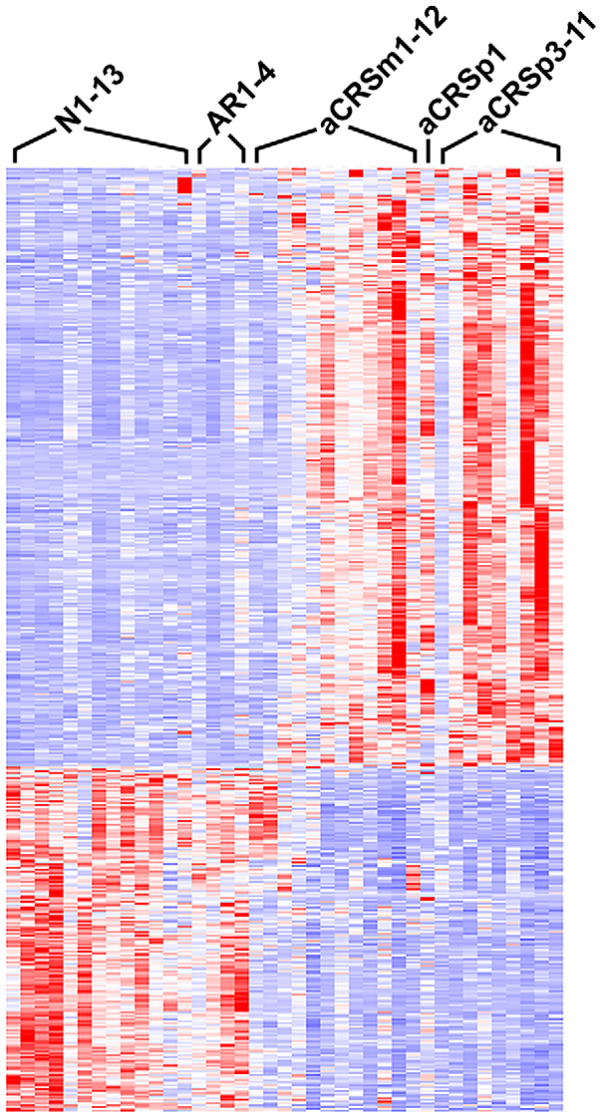
Heat Map Profiles of the 39 Nasosinus Tissue Specimens Analyzed by Exon Array. Each horizontal bar represents 1 of the 528 genes identified as differentially transcribed between clearcut Normal samples (N1 to N8) and clearcut aCRS polyp samples (aCRSp3 to aCRSp11). The bar color indicates the relative transcription level from blue (low) through white to red (high). Inflamed mucosa samples are indicated by “m”, and aCRSm12 was a small sample of mixed inflamed mucosa and polyp. The inflamed mucosa (m) and polyp (p) aCRS samples with the same ending number are from the same donor.

Demographic and clinical data for the 12 aCRS and the 17 normal or rhinitis subjects donating tissue for exon array analyses are shown in Table S1. Besides focusing on CRS patients with concurrent asthma, most of whom also had nasal polyposis, relatively young subjects (18 to 58 years old) were recruited to focus on immune-related rather than potential age-related nasosinus tissue changes. To assess the clinical categorization of the various subjects' specimens (i.e. normal, rhinitis, aCRS inflamed mucosa, or aCRS polyp), data from the most phenotypically distinct specimens, 9 clearcut aCRS nasal polyp (aCRSp3 through aCRSp11) and 8 clearcut normal (N1 through N8) specimens, were compared using a gene-level analysis of ∼22,000 well-established genes. This preliminary comparison identified 528 putative differentially-transcribed genes with Fc>2.0 and adjusted p-value<0.05 (unpaired nonparametric test with Benjamini-Hochberg False Discovery Rate adjustment). These genes were then used to assess where the remaining specimens fell in comparison (Figure 1). The normal and rhinitis samples were grouped together relative to all but two of the aCRS inflamed mucosa samples (aCRSm1 and aCRSm2). These two samples had mixed heat map profiles (Figure 1), with some sections similar to the normal and rhinitis samples and other sections more like the other aCRS samples (both inflamed mucosa and polyp). Interestingly, one of these samples (aCRSm2) was from the one aCRS patient without nasal polyps.

Given the distinct transcript profile for most of the 22 aCRS samples, this group of samples (designated aCRSwNP; 20 samples of inflamed mucosa, polyp, or both from 11 aCRSwNP patients, with the aCRSm1 and aCRSm2 samples excluded), was compared to all of the normal and rhinitis samples (n = 17). An analysis considering only well-established genes identified 447 differentially-transcribed genes (261 with increased transcription) at Fc>2.0 and adjusted p-value<0.05 (Table 1 and Table S2; to identify the data for a particular gene in Table S2 use the “Find” function to search using any Gene Symbol mentioned in this article, e.g. “CCL8” or “CRISP2”). Notably, several genes in Table 1 and Table S2 were also identified by Stankovic et al. in their aspirin-sensitive aCRSwNP patients [11]. These included increased transcription of periostin (POSTN) and DC-SIGN (CD209) and decreased transcription of zinc alpha-2-glycoprotein 1 (AZGP1), statherin (STATH), and prolactin-induced protein (PIP).

**Table 1:** Top 10 over- and under-transcribed genes in asthmatic chronic rhinosinusitis with nasal polyps (aCRSwNP).

Transcript ID	Fold Change	p-value*	gene_assignment
3718902	76.43	5.43E-15	NM_002988 // CCL18 // chemokine (C-C motif) ligand 18 // 17q11.2 // 6362
3862108	32.69	1.63E-13	NM_001828 // CLC // Charcot-Leyden crystal protein // 19q13.1 // 1178
3901361	31.53	1.82E-11	NM_001898 // CST1 // cystatin SN // 20p11.21 // 1469
3718204	14.01	1.30E-12	NM_005408 // CCL13 // chemokine (C-C motif) ligand 13 // 17q11.2 // 6357
2497082	8.82	1.91E-11	NM_016232 // IL1RL1 // interleukin 1 receptor-like 1 // 2q12 // 9173
3901387	8.34	3.19E-10	NM_001322 // CST2 // cystatin SA // 20p11.21 // 1470
3510066	7.89	1.19E-13	NM_006475 // POSTN // periostin, osteoblast specific factor // 13q13.3 // 10631
3818596	7.69	1.38E-10	NM_001974 // EMR1 // egf-like module containing, mucin-like, hormone receptor-like 1 // 19p13.3 // 2015
3852832	6.82	1.12E-11	NM_032571 // EMR3 // egf-like module containing, mucin-like, hormone receptor-like 3 // 19p13.1 // 84658
2940202	6.77	5.38E-13	NM_000129 // F13A1 // coagulation factor XIII, A1 polypeptide // 6p25.3-p24.3 // 2162
3063589	−6.66	6.31E-07	NM_001185 // AZGP1 // alpha-2-glycoprotein 1, zinc // 7q22.1 // 563
2923270	−6.99	9.77E-11	NM_002667 // PLN // phospholamban // 6q22.1 // 5350
2730173	−7.32	1.08E-05	NM_003154 // STATH // statherin // 4q11–q13 // 6779
2489007	−7.38	1.14E-10	NM_001615 // ACTG2 // actin, gamma 2, smooth muscle, enteric // 2p13.1 // 72
3728637	−7.70	6.29E-09	NM_006151 // LPO // lactoperoxidase // 17q23.1 // 4025
2730431	−10.25	3.79E-08	NM_152291 // MUC7 // mucin 7, secreted // 4q13–q21 // 4589
3028934	−16.93	1.26E-09	NM_002652 // PIP // prolactin-induced protein // 7q34 // 5304
3444578	−17.50	6.46E-06	NM_002723 // PRB4 // proline-rich protein BstNI subfamily 4 // 12p13.2 // 5545
2925013	−18.67	1.73E-09	NM_001010905 // C6orf58 // chromosome 6 open reading frame 58 // 6q22.33 // 352999
2956563	−28.15	3.85E-13	NM_006061 // CRISP3 // cysteine-rich secretory protein 3 // 6p12.3 // 10321

*unpaired nonparametric test with Benjamini-Hochberg False Discovery Rate adjustment.

**Figure 2:**
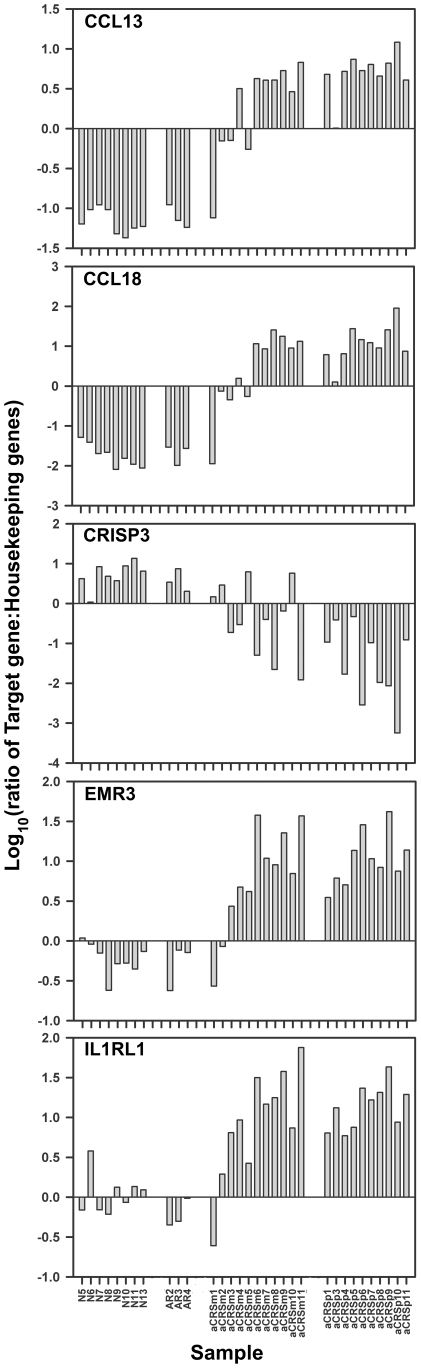
Relative Transcription Levels of Selected Genes by qRT-PCR. The ratio of the relative quantity of the designated target gene (CCL13, CCL18, CRISP3, EMR3, or IL1RL1) to that of one of three housekeeping genes (B2M, ACTB, or GAPDH) was determined. Because each of the three housekeeping genes gave proportionally similar ratio results, an average target gene to housekeeping genes ratio was calculated and plotted for each of the individual samples indicated along the x-axis [normal (n = 8), AR (n = 3), aCRS without nasal polyps (n = 1; aCRSm2), and aCRSwNP (n = 10, including both inflamed mucosa and polyp tissue samples from each of these 10 subjects)].

Transcription levels of five of the identified genes, which were also among 221 genes identified as differentially transcribed when comparing only aCRS inflamed mucosa (n = 11; aCRSm1 through aCRSm11) to normal and rhinitis (n = 17) exon array data (data not shown), were assessed by qRT-PCR (Figure 2). In agreement with the exon array data, CCL13, CCL18, EMR3, and IL1RL1 transcription was increased while CRISP3 transcription was decreased in inflamed mucosa and polyp tissue from aCRSwNP subjects.

**Figure 3:**
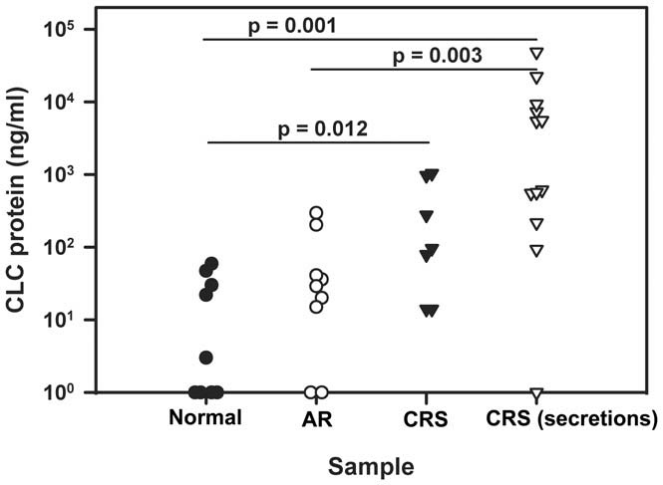
Charcot-Leyden Crystal (CLC) Protein Levels in Various Nasosinus Lavage Fluids or Secretions. Nasal lavage fluid was collected from subjects distinct from those donating tissue for exon array analyses and with no history of nasosinus disease (n = 9, Normal), with active allergic rhinitis (n = 9, AR), and with asthmatic CRS (n = 7, CRS). Nasal secretions were also collected by gentle suction from additional subjects diagnosed with CRS (n = 12, CRS(secretions)). Each sample was assayed for CLC protein by a competitive radioimmunoprecipitation assay.

Because mRNA levels are not always directly proportional to their corresponding protein levels, we utilized an immunoassay available in our laboratory to assess CLC levels in nasal lavage fluid or suction-collected secretions from a separate population of CRS subjects. This showed significantly elevated CLC protein in CRS specimens relative to nasal lavage fluids from normal controls or AR subjects with active disease (Figure 3), similar to the observed CLC mRNA increase in aCRSwNP (Table 1). We also recently reported increased CCL18 protein levels in induced sputum from asthmatic subjects [17], which is consistent with the increased CCL18 transcription observed here in aCRSwNP. Overall, comparisons with a similar array study of aspirin-sensitive aCRSwNP [11] and several assays at both the mRNA and protein levels support our gene-level exon array results.

**Figure 4:**
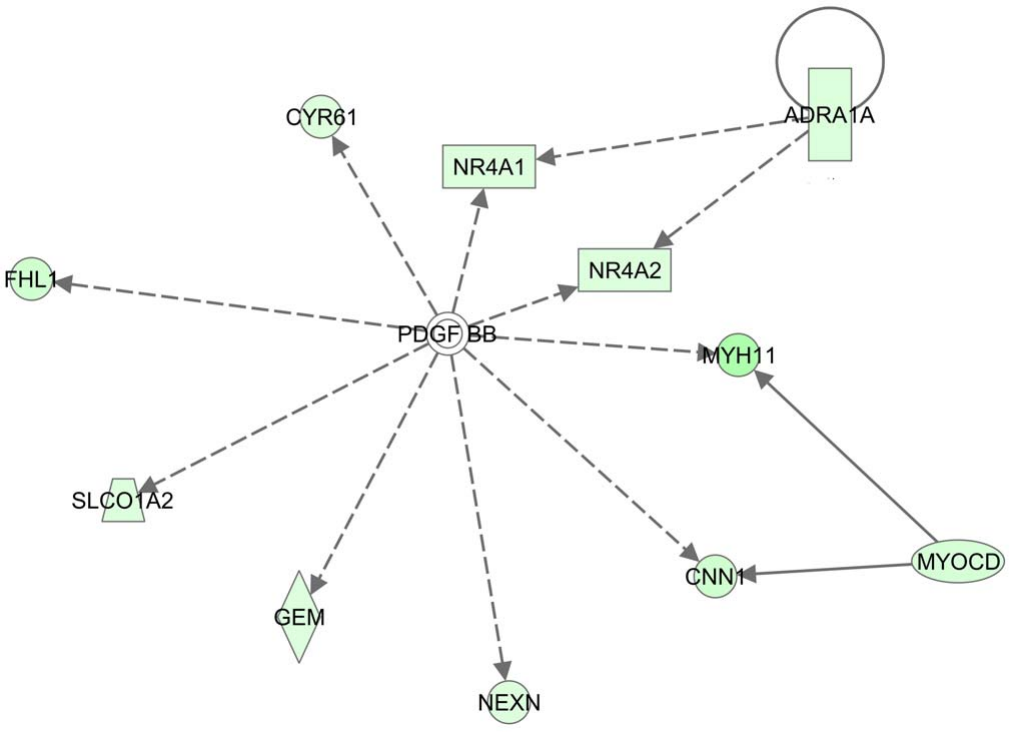
Molecular Network Implicating Platelet-Derived Growth Factor Beta Homodimer (PDGF BB) in aCRSwNP Pathology. The genes showing reduced transcription in aCRSwNP (see Table S2) were analyzed using Ingenuity Pathways Analysis software, and this was the first-most significant network identified. The intensity of green indicates the degree of reduced transcription, and the length of a line reflects the evidence supporting the relationship between two genes – shorter lines indicating stronger literature support. The geometric shape associated with a gene symbol indicates the gene product's function. ADRA1A, alpha-1A-adrenergic receptor; CNN1, basic smooth muscle calponin 1; CYR61, cysteine-rich angiogenic inducer 61; FHL1, four and a half LIM domains 1; GEM, GTP binding protein overexpressed in skeletal muscle; MYH11, smooth muscle myosin heavy chain 11; MYOCD, myocardin; NEXN, nexilin (F actin binding protein); NR4A1, nuclear receptor subfamily 4, group A, member 1; NR4A2, nuclear receptor subfamily 4, group A, member 2; SLCO1A2, solute carrier organic anion transporter family, member 1A2.

Because molecular pathway or network analyses can identify biologically relevant gene interactions that might be hidden when considering only individual genes, Ingenuity Pathways Analysis software was used to analyze the 447 differentially-transcribed genes from the comparison between aCRSwNP and normal/rhinitis samples. In general, the top canonical pathways included those related to leukocyte extravasation, hepatic fibrosis, eicosanoid signaling, chemokine signaling, IL10 signaling, and the complement system. The presumed inflammation-associated networks involving NF-kB, Integrins, IL6, TGFb, and TNF family members and Th2-associated networks involving IL4 and IL13 were among the non-canonical molecular networks identified. A down-regulated PDGFB homodimer-centric network involving several smooth muscle-related genes was also identified (Figure 4). In parallel analyses, differentially transcribed genes in a second eosinophilic inflammatory disease, AD, were compared to those in aCRSwNP to identify common eosinophil-associated inflammatory pathways, including those likely occurring early in the inflammatory process [16]. Table 2 shows a list of 85 common genes and their tentative functional groupings. Interestingly, nine genes associated with smooth muscle, including several in the PDGFB network implicated by our aCRSwNP data, also showed decreased transcription in AD (Table 2, Group 1).

**Table 2:** Genes Showing Similar Differential Transcription in aCRSwNP and AD.

Gene Symbol	RefSeq ID	Potential Group*	Gene Symbol	RefSeq ID	Potential Group	Gene Symbol	RefSeq ID	Potential Group
**Decreased Transcription**								
ACTG2**	NM_001615	1	**MYH11**	NM_002474	1	PRKAA2	NM_006252	
CNN1	NM_001299	1	MYRIP	NM_015460	1	PRR4	NM_007244	
DES	NM_001927	1	NAP1L2	NM_021963		RNF128	NM_194463	
**FABP4** ***	NM_001442		NPY1R	NM_000909		SORBS1	NM_015385	
FHL1	NM_001449	1	OGN	NM_033014		TAGLN	NM_001001522	1
GPM6A	NM_201592		PIP	NM_002652	2	TSPAN6	NM_003270	
**LMOD1**	NM_012134	1	**PLN**	NM_002667	1			
**MSMB**	NM_138634		PRB4	NM_002723				
**Increased Transcription**								
ADAM8	NM_001109	2	GNA15	NM_002068		PIM2	NM_006875	
ALOX15	NM_001140		GPR109B	NM_006018		**PTPRC**	NM_080922	
ALOX5AP	NM_001629		GPR44	NM_004778	5	RAC2	NM_002872	
ARRB2	NM_004313		HRH4	NM_021624	5	RHOH	NM_004310	
**CCL13**	NM_005408	3	IFI30	NM_006332		**S100A8**	NM_002964	
**CCL18**	NM_002988	3	IGFBP3	NM_001013398		**S100A9**	NM_002965	
**CCL2**	NM_002982	3	IL1RL1	NM_016232	5	SAMSN1	NM_022136	
**CCL22**	NM_002990	3	**IL4R**	NM_000418	5	**SELE**	NM_000450	6
CCL26	NM_006072	3	ITGAM	NM_000632	6	**SELPLG**	NM_003006	6
CCR1	NM_001295	3	KIAA1199	NM_018689		**SLA**	NM_006748	
**CD1B**	NM_001764	4	LCP1	NM_002298		**SLAMF8**	NM_020125	
**CD1C**	NM_001763	4	LCP2	NM_005565		**SLC7A8**	NM_012244	
**CD1E**	NM_030893	4	LST1	NM_205837		SPON1	NM_006108	
CD300A	NM_007261	5	LTB	NM_009588		**SRGN**	NM_002727	
**CD52**	NM_001803		MARCO	NM_006770		SYK	NM_003177	
CLEC4A	NM_194450		**MMP9**	NM_004994	2	TGFB1	NM_000660	3
**CTSC**	NM_148170	2	MNDA	NM_002432		TMEM156	NM_024943	
CXCL13	NM_006419	3	**MS4A4A**	NM_024021	5	**TNC**	NM_002160	
DOCK2	NM_004946		MXD1	NM_002357		**TOP2A**	NM_001067	
**DOK2**	NM_003974		NP	NM_000270				
FFAR2	NM_005306		PCSK1	NM_000439	2			
FPR3	NM_002030		PCSK6	NM_138319	2			

*1 = muscle-related; 2 = protease/protease-like; 3 = chemokine or cytokine; 4 = antigen presentation; 5 = allergy-associated receptor; 6 = cell adhesion.

**For information about any of the listed genes, go to the National Center of Biotechnology Information (NCBI) website and search using the gene's Gene Symbol (e.g. ACTG2) or RefSeq ID (e.g. NM_001615).

***Genes in bold showed similar and significant differential transcription in both of the atopic dermatitis data sets used.

## Discussion

Because aCRSwNP and other eosinophilic epithelial diseases are prevalent and difficult to control, early and specifically-targeted interventions against eosinophilic inflammation are needed. Therefore, we gathered aCRSwNP array data and compared it to other reported array data. Overall, we verified the differential expression of several genes recently associated with aspirin-sensitive aCRSwNP [11] and implicated several other genes or gene sets in the earlier stages of eosinophilic inflammation.

Assessing the validity of our exon array data at the entire gene transcript level was an initial goal. Comparison to the recent Stankovic et al. study [11], involving a patient population similar to ours and using more anatomically matched normal control tissue (i.e. 7 ethmoid sinus specimens of the 10 nasosinus control specimens), provided confidence because differential expression of several genes important in aspirin-sensitive aCRSwNP were also identified in our data (decreased AZGP1, STATH, and PIP and increased DC-SIGN (CD209) and POSTN; with increased POSTN expression also being observed in eosinophilic esophagitis (EE) [18]). Interestingly, these results were often opposite to those of Liu et al., who reported increased transcription of AZGP1, STATH, PIP, and CRISP3 [10]. The most apparent reason for these differences may be the required use of intranasal glucocorticoids for at least 1 month before tissue collection in the Liu et al. study [10]. Our qRT-PCR results for CCL13, CCL18, CRISP3, EMR3, and IL1RL1 (ST2) (Figure 2) and the increased presence of CLC (Figure 3) and CCL18 [17] protein in separately collected airway specimens from CRS or asthmatic patients, in accordance with their increased mRNA levels in our aCRSwNP patients, further support the validity of our exon array data. Notably, none of the five qRT-PCR-confirmed genes (Figure 2) have been specifically associated with aCRSwNP before and their differential transcription in inflamed mucosa potentially suggests their involvement in the earlier stages of inflammation.

Transcript levels of the five qRT-PCR-confirmed genes in aCRSwNP were relatively distinct when compared to the AR samples (Figure 2). However, the mild disease of the AR tissue donors (because they chose to undergo nasosinus surgery outside of their active allergy season) could suggest that the differences between aCRSwNP and AR were caused by differences in disease severity rather than disease-specific gene transcript profiles. Still, a study of AR without and with nasal polyps indicated an association of mammaglobin-1 and 33 other genes with nasal polyposis [19]. However, neither mammaglobin-1 nor a substantial majority of the other 33 genes (only increases in prostaglandin D2 synthase and cystatin S appeared common) were among the genes showing differential transcription in our aCRSwNP samples. Thus, it remains possible that gene transcript profiles unique or only partially overlapping between AR and aCRSwNP exist, with nasal polyposis in these two diseases mediated by two separate mechanisms.

While several other putative differentially transcribed genes or gene sets identified here are likely of interest (e.g. cystatins, osteopontin (SPP1), histamine H4 receptor, etc.; Table S2), we are limiting our further discussion to gene sets that appear relevant to the eosinophilic inflammation of aCRSwNP and other eosinophilic epithelial diseases.

The most prominent class of up-regulated genes identified in aCRSwNP appeared to be the chemokines (Table S2). The strong eosinophil chemoattractants eotaxin-1 (CCL11), −2 (CCL24), and −3 (CCL26) (the latter also increased in AD (Table 2) and EE [20]) were up-regulated. Several other up-regulated genes that are adjacent to CCL11 at chromosome 17q11.2 included CCL13 (MCP4), CCL2 (MCP1), and CCL8 (MCP2); both CCL13 and CCL2 were also up-regulated in AD skin (Table 2). Thus, induction of this 17q11.2 gene cluster likely contributes strongly to eosinophilic epithelial inflammation, and the 17q11.2 locus was previously identified as a candidate gene region associated with AD [21]. When analyzed for relevant molecular networks, the increased transcription of most of these chemokines and some of their receptors in aCRSwNP shows an association to NF-kB-mediated regulation (Figure S1).

Both aCRSwNP and AD showed increased transcription of CCL18 (PARC), CCL22 (MDC), and CXCL13. These are notable because of their production from IL4- or IL13-“alternatively activated” members of the mononuclear phagocyte system, which includes monocytes, macrophages, and dendritic cells [22], [23]. So-called inflammatory dendritic cells (also known as inflammatory dendritic epidermal cells (IDECs)) likely represent a prominent form of CD11c+ monocyte-derived alternatively-activated dendritic cells in AD skin [24], [25], and an abundant CD11c+ cell population can also be observed in CRS with nasal polyposis (Figure S2). Other cell types, such as epithelial cells, may also produce these chemokines under these conditions. CCL18 and CCL22 are associated with chemoattraction of Th2 cells, which further amplify eosinophilic inflammation. The CCL18 gene is also located near the eotaxin-1 and MCP genes cluster at chromosome 17q11.2. Intriguingly, these genes are substantially up-regulated 4 hours after allergen inhalation challenge in Ascaris suum-sensitized monkeys with marked post-challenge eosinophil lung infiltration [26], and CCL18 and CCL13 appear to be up-regulated even in acute or minimally lesional AD skin [27], [16], [25]. An alternative activation profile is also observed in severe asthma exacerbations [28]. Thus, the 17q11.2 genes likely contribute relatively early to the eosinophilic inflammatory response.

IL1RL1 (ST2) was recently identified as the receptor for IL33 (a member of the IL1 family, although the IL33 gene is located on a separate chromosome). This receptor-ligand pair may contribute to both innate and acquired Alternaria-induced eosinophil responses of the airways via Alternaria-induced IL33 release from airway epithelial cells (H. Kouzaki & H. Kita, unpublished data) and via expression of IL1RL1 directly by eosinophils and indirectly by basophils, mast cells, dendritic cells, and Th2 cells [29], [30], [31]. Increased IL1RL1 transcription is also observed in AD (Table 2), and IL33 appears to be increased in AD skin and anaphylactic responses [32]. IL33 amplification of IL13-induced alternatively-activated macrophage polarization in asthma patients has also recently been suggested [33]. Thus, the IL33-IL1RL1 interaction is likely a key contributor to both innate and acquired mechanisms of eosinophilic inflammation in aCRSwNP.

Increased transcription of both EMR3 and EMR1 was observed in our exon array data. These appear to be G protein-coupled seven-transmembrane receptors, and among peripheral blood leukocytes, EMR1 was expressed predominantly by eosinophils [34]. The mucin-like domain of these molecules further suggests that they contribute to eosinophil adhesion and activation. Whether EMR3 and EMR1 transcription per eosinophil is increased in aCRSwNP or the increased transcription reflects elevated numbers of infiltrating eosinophils is unknown. Regardless, these receptors will likely help mediate eosinophil function that contributes to disease pathology. In contrast to EMR3 and EMR1, our exon array data showed decreased transcription of CRISP3 and CRISP2. Although the function(s) of these molecules is obscure, decreased transcription of CRISP3 is observed in EE and associated with the presence of IL13 [20].

Decreased expression of S100A8 and S100A9, members of the epidermal differentiation complex at chromosome 1q21, in epithelial cells from CRS patients with and without nasal polyps was recently reported [35], [36]. However, our analyses indicate increased transcription of S1008A and S100A9 in whole tissue from both aCRSwNP and AD patients (Table 2), and in a proteomics study, we detected both S1008A and S1009A in nasal lavage fluids from aCRS subjects [37]. This apparent discrepancy might be explained by a compensatory increase in S100A8 and S100A9 expression by a non-epithelial cell type, as suggested by Tieu et al. [36], but this awaits further clarification.

Analysis of the differentially transcribed genes in aCRSwNP using Ingenuity Pathway Analysis software identified disease-associated networks related to inflammation (involving molecules like NF-kB, IL6, TNF, and TGFb1), cell adhesion (ITGB2 and ITGAM), and Th2-type cytokines (IL4 and IL13), and IL4R is increased in both AD and aCRSwNP (Table 2). Interestingly, a molecular network centered around the homodimer of PDGFB (i.e. PDGFBB) included a series of genes showing decreased transcription in aCRSwNP (Figure 4). Furthermore, several genes within this network (MYH11, CNN1, FHL1), and several other genes related to smooth muscle (ACTG2, DES, LMOD1, MYRIP, PLN, and TAGLN), were also differentially transcribed in AD (Table 2). How decreased transcription of these genes might contribute to disease pathology is unclear. However, a PDGFRA mutation was associated with Gleevac-sensitive hypereosinophilic syndrome (HES), so perhaps decreased PDGFB-related transcription is also associated with an eosinophilic response. Alternatively, perhaps the epithelial hyperreactivity or altered vascular tone common to eosinophilic diseases and involving smooth muscle control are influenced by PDGFB-associated gene expression. Regardless, our results are the first indication that decreased gene expression of several smooth muscle-related genes associated with PDGFB may be involved in the pathology of aCRSwNP and other eosinophilic epithelial diseases.

**Figure 5:**
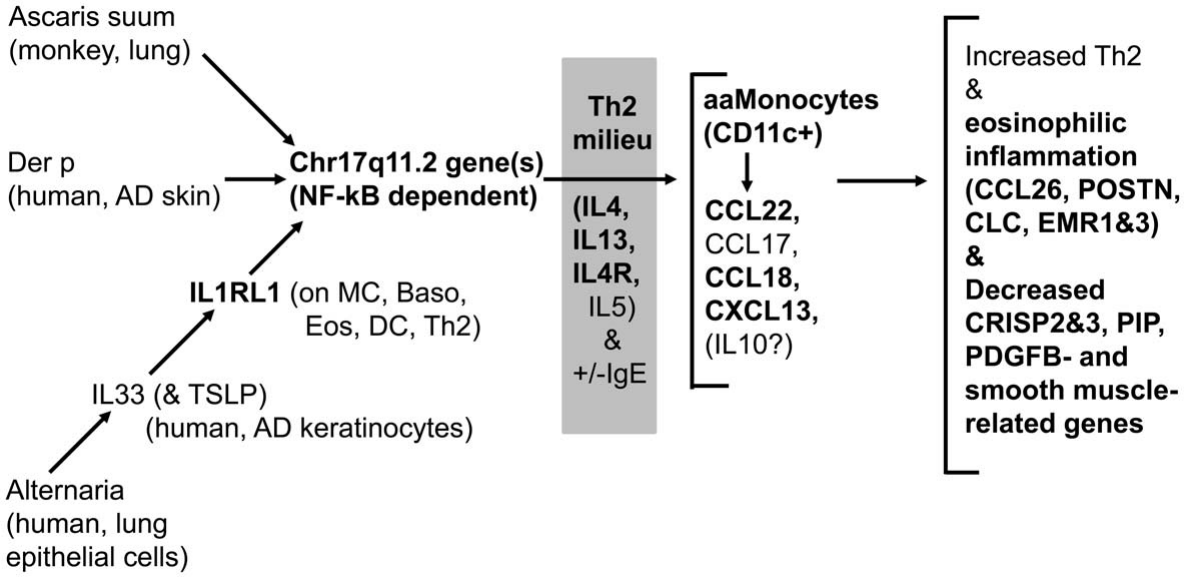
Potential Relationship of Observed Gene Transcription Changes with Eosinophilic Inflammation. Three stimuli (Ascaris suum, Dermatophagoides pteronyssinus (Der p), and Alternaria) associated with eosinophilic inflammation appear to induce one or more of the Chromosome 17q11.2 eotaxin-1 (CCL11) or MCP genes [25], [26]. Alternaria may induce this gene transcription via the IL33 – IL1RL1 interaction, and this interaction appears to contribute to eosinophilic inflammation of human AD and aCRSwNP, in part via an NFkB-dependent mechanism [32]. In the Th2-like milieu of AD and aCRSwNP, likely arising from an unfavorable mix of environmental exposures and genetic susceptibilities, alternatively-activated monocyte-derived cells (aaMonocytes), such as “inflammatory DCs” [24], may develop and amplify the Th2-like eosinophilic response via chemokine production. Other genes likely associated with Th2-like eosinophilic inflammation are also indicated. Items in bold are supported by our array data. MC, mast cell; Baso, basophil; Eos, Eosinophil; DC, dendritic cells.

In summary, exon array data for adjacent inflamed mucosa and polyp tissue from aCRSwNP patients confirmed the differential transcription of several genes that appear important in aspirin-sensitive aCRSwNP [11]. It also identified several genes or gene sets that likely contribute to the earlier stages of the eosinophilic inflammation of aCRSwNP (Figure 5), and some of these genes are also differentially transcribed in other eosinophilic epithelial diseases, such as AD and EE. Overall, these data should assist in the pursuit of improved treatments for aCRSwNP and other eosinophilic diseases.

## Supporting Information

Table S1 Nasosinus Tissue Donor Characteristics.
(0.10 MB DOC)

Table S2 Differentially Transcribed Genes in Asthmatic Chronic Rhinosinusitis with Nasal Polyposis.
(0.13 MB XLS)

Figure S1 Molecular Network Implicating Nuclear Factor-kappa B (NF-kB) in the Increased Transcription of aCRSwNP-associated Chemokines/Chemokine Receptors and Other Genes. The genes showing increased transcription in aCRSwNP (see Table S2) were analyzed using Ingenuity Pathways Analysis software, and this was the first-most significant network identified. The intensity of red indicates the degree of increased transcription.
(1.30 MB TIF)

Figure S2 Increased CD11c+ Cells in CRS with Nasal Polyposis. Nasosinus tissue from a CRS patient with nasal polyps (A) or from a normal control (B) was stained with anti-CD11c. The presence of CD11c is shown by reddish-brown staining.
(5.71 MB TIF)
