# Supplementary material for: Gene Transcription Changes in Asthmatic Chronic Rhinosinusitis with Nasal Polyps and Comparison to Those in Atopic Dermatitis
Source: PLoS One. 2010 Jul 6;5(7):e11450. doi: 10.1371/journal.pone.0011450 (PMC2897889; doi:10.1371/journal.pone.0011450)
Supplement: Table S1 — Nasosinus Tissue Donor Characteristics. (0.10 MB DOC) [file pone.0011450.s001.doc]

| **Supplemental Table 1: Nasosinus Tissue Donor Characteristics. (DA Plager et al., Mayo Clinic, Rochester, Minnesota, USA)** | | | | | | | | | |
| --- | --- | --- | --- | --- | --- | --- | --- | --- | --- |
| **Donor ID** | **Exon Array**  **.CEL file ID** | **Age**  **(yrs)** | **Sex** | **Race** | **Asthma** | **IgE@** | **5-part WBC***  **(Eo%, Eo# x 10(9)/L;**  **WBC abnormalities)** | **Aspirin**  **sensitive** | **Comments** |
| **Normal** |  |  |  |  |  |  |  |  |  |
| N1 | Pla032HuEx | 22 | F | White | No | – | 1.4%, 0.1 | No |  |
| N2 | Pla034HuEx | 22 | M | White | No | – | 2.0%, 0.1 | No |  |
| N3 | Plager23HuEx | 42 | M | White | No | – | 7.9%, 0.47 | No |  |
| N4 | Plager25HuEx | 26 | F | White | No | SRW-C2 | 0.3%, 0.02 (low) | No |  |
| N5 | Pla120HuEx | 29 | M | White | No | – | 3.3%, 0.25 | No |  |
| N6 | Pla098HuEx | 26 | F | White | No | – | 0.8%, 0.05 | No |  |
| N7 | Pla101HuEx | 55 | M | White | No | – | 8.9%, 0.66 (high) | No |  |
| N8 | Pla102HuEx | 56 | M | White | No | – | 2.1%, 0.12 | No |  |
| N9 | Pla059HuEx | 29 | F | White | No | – | 0.6%, 0.05 | No | Mother with CRS |
| N10 | Pla063HuEx | 37 | M | Hispanic | No | HDM-C1; TG-C2 | 2.5%, 0.11 | No | AR? |
| N11 | Pla097HuEx | 21 | F | White | No | – | 1.0%, 0.05 | No | Vasomotor rhinitis? |
| N12 | Pla103HuEx | 26 | M | White | No | – | 4.9%, 0.26;  Ly% 51.3 (high) | No | AR? |
| N13 | Pla031HuEx | 51 | M | White | No | HDM-C1 | 0.7%, 0.05 | No | Diabetic |
| **AR** |  |  |  |  |  |  |  |  |  |
| AR1 | Pla033HuEx | 19 | F | White | No | – (Rat SPT+) | 6.0%, 0.45 | No |  |
| AR2 | Pla104HuEx | 20 | F | White | No | Alt-C3; Cat-C4; SRW-C2 | N/A | No |  |
| AR3 | Pla121HuEx | 37 | M | White | No | Alt-C2; SRW-C4 | 1.8%, 0.14 | No |  |
| AR4 | Pla064HuEx | 18 | F | N/A | No | – | 1.6%, 0.09 | N/A | Asthma? |
| **CRS** |  |  |  |  |  |  |  |  |  |
| aCRSm1 | Pla037HuEx | 25 | F | White | Yes | Alt-C3; HDM-C3 | 3.3%, 0.2 | No |  |
| aCRSp1 | Plager19HuEx | “ | “ | “ | “ | “ | “ | “ |  |
| aCRSm2 | Pla124HuEx | 58 | M | Asian | Yes# | – | 5.5%, 0.44 | No |  |
| aCRSm3 | Pla065HuEx | 23 | F | N/A | Yes# | – | 2.7%, 0.16 | N/A |  |
| aCRSp3 | Pla100HuEx | “ | “ | “ | “ | “ | “ | “ |  |
| aCRSm4 | Pla125HuEx | 36 | M | White | Yes | HDM-C3; SRW-C1; TG-C2 | 6.1%, 0.35 | Yes |  |
| aCRSp4 | Pla119HuEx | “ | “ | “ | “ | “ | “ | “ |  |
| aCRSm5 | Pla126HuEx | 45 | F | White | Yes | – | 12.6%, 1.01 (high) | Yes |  |
| aCRSp5 | Pla061HuEx | “ | “ | “ | “ | “ | “ | “ |  |
| aCRSm6 | Pla060HuEx | 47 | M | White | Yes | HDM-C2 | 0.2%, 0.04 (low);  Ne# 15.6 x 10^9^/L (high)  Mo# 1 x 10^9^/L (high)  Ly% 5.5% (low) | No |  |
| aCRSp6 | Plager21HuEx | “ | “ | “ | “ | “ | “ | “ |  |
| aCRSm7 | Pla099HuEx | 45 | F | White | Yes | – (horse hair?) | 4.9%; 0.4 | No |  |
| aCRSp7 | Pla066HuEx | “ | “ | “ | “ | “ | “ | “ |  |
| aCRSm8 | Pla105HuEx | 25 | M | White | Yes | Cat-C1; SRW-C2 | N/A | No |  |
| aCRSp8 | Pla106HuEx | “ | “ | “ | “ | “ | “ | “ |  |
| aCRSm9 | Pla122HuEx | 24 | F | White | Yes | N/A | N/A | Yes |  |
| aCRSp9 | Pla123HuEx | “ | “ | “ | “ | “ | “ | “ |  |
| aCRSm10 | Pla036HuEx | 45 | M | White | Yes | – | 8.1%, 0.65 (high)  Ly% 19.2 (low) | Yes |  |
| aCRSp10 | Pla062HuEx | “ | “ | “ | “ | “ | “ | “ |  |
| aCRSm11 | Pla067HuEx | 41 | F | White | Yes | Cat-C2 | N/A | Yes |  |
| aCRSp11 | Pla068HuEx | “ | “ | “ | “ | “ | “ | “ |  |
| aCRSm12 | Pla038HuEx | 41 | F | White | Yes | – | 0.2%, 0.04 (low);  Ne# 12.7 x 10^9^/L(high)  Mo# 1.1 x 10^9^/L (high)  Ly% 8.9 (low) | No | Mixed mucosa & polyp sample |
| * AR = allergic rhinitis, aCRS = asthmatic chronic rhinosinusitis, m=inflamed mucosa; p=polyp; N/A = Not available; SPT = skin prick test; WBC = White Blood Cell count (Neutrophils (Ne); Lymphocytes (Ly); Monocytes (Mo); Eosinophils (Eo); Basophils (Ba))  ^#^ asthma diagnosis based primarily on a positive bronchial methacholine challenge test  @ serum RAST test results for five common allergens: Alternaria (Alt), Cat Epithelium (Cat), House dust mite (HDM; Der F), Short Ragweed (SRW), and Timothy Grass (TG); Negative (–) <0.35, Class 1(C1)=0.35-0.70, C2=0.71-3.50, C3=3.51-17.5 , C4=17.6-50, C5=50.1-100 kU/L | | | | | | | | | |
