# Supplementary figures and images for: Gene Transcription Changes in Asthmatic Chronic Rhinosinusitis with Nasal Polyps and Comparison to Those in Atopic Dermatitis
Source: PLoS One. 2010 Jul 6;5(7):e11450. doi: 10.1371/journal.pone.0011450 (PMC2897889; doi:10.1371/journal.pone.0011450)

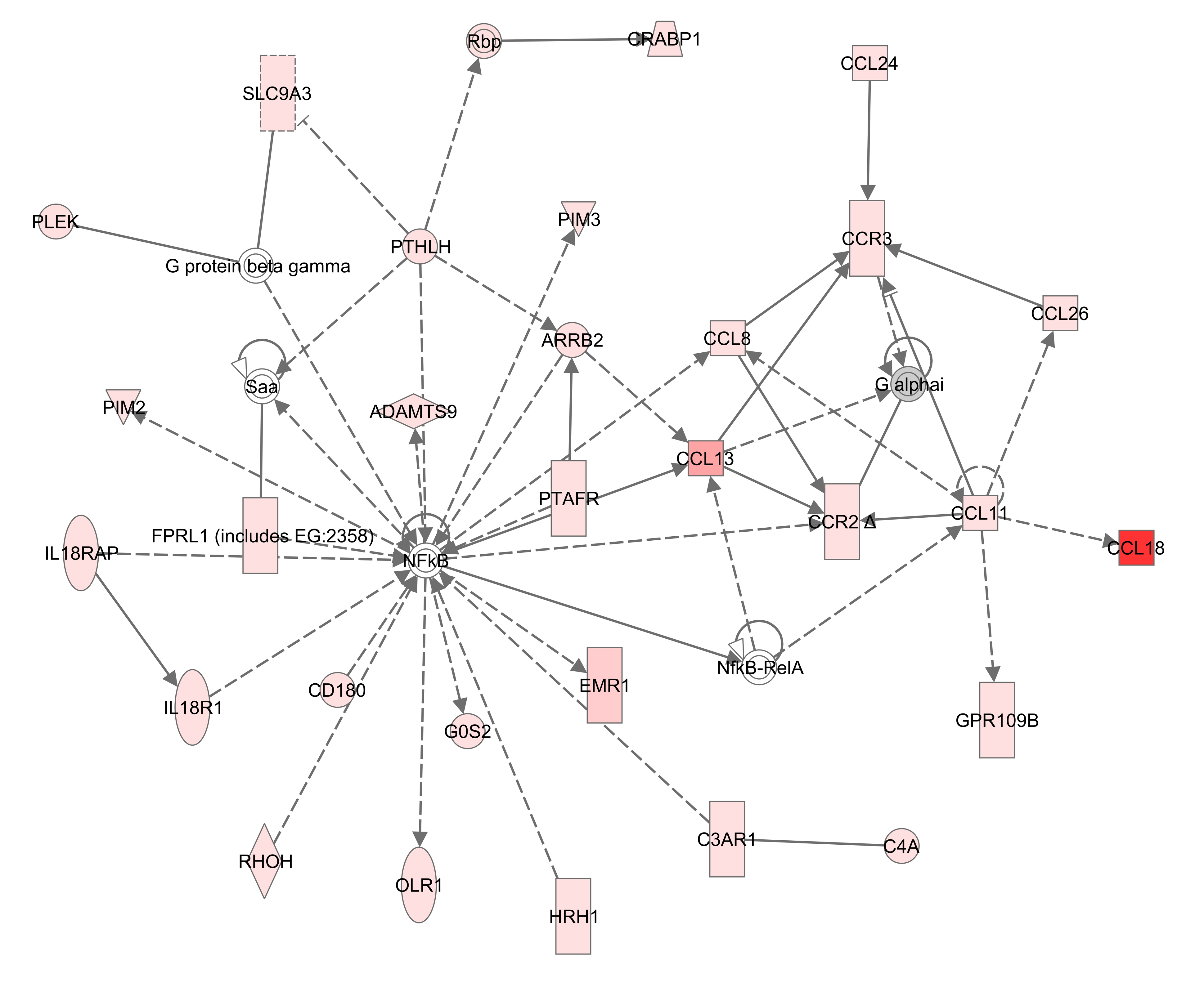

Supplement: Figure S1 — Molecular Network Implicating Nuclear Factor-kappa B (NF-kB) in the Increased Transcription of aCRSwNP-associated Chemokines/Chemokine Receptors and Other Genes. The genes showing increased transcription in aCRSwNP (see Table S2) were analyzed using Ingenuity Pathways Analysis software, and this was the first-most significant network identified. The intensity of red indicates the degree of increased transcription. (1.30 MB TIF) [file pone.0011450.s003.tif]

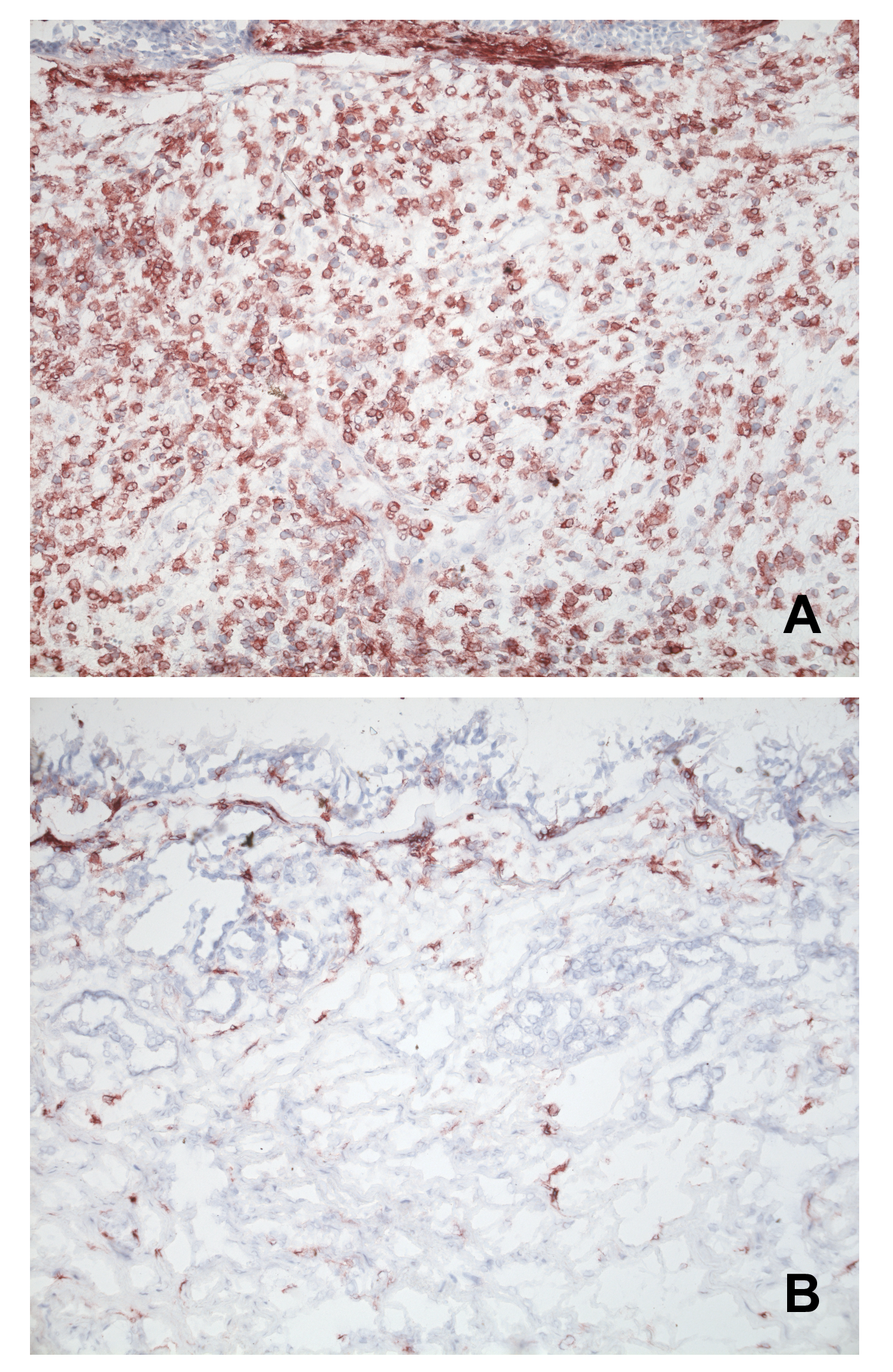

Supplement: Figure S2 — Increased CD11c+ Cells in CRS with Nasal Polyposis. Nasosinus tissue from a CRS patient with nasal polyps (A) or from a normal control (B) was stained with anti-CD11c. The presence of CD11c is shown by reddish-brown staining. (5.71 MB TIF) [file pone.0011450.s004.tif]
